# Effects of one-hour daily outdoor access on milk yield and composition and behaviors of tethered dairy cows

**DOI:** 10.5713/ab.23.0539

**Published:** 2024-05-07

**Authors:** Huricha, Ai Nanbu, Masashi Takemoto, Ken-ichi Takeda

**Affiliations:** 1 Faculty of Agriculture, Shinshu University, Kamiina, Nagano 399-4598, Japan; 2 Present address: Western Region Agricultural Research Center, National Agriculture and Food Research Organization (NARO), Shimane 694-0013, Japan; 3 Livestock Research Institute, Forestry and Fisheries Research Center, Toyama Prefectural Agricultural, Toyama 939-2622, Japan; 4 Institute of Agriculture, Academic Assembly, Shinshu University, Kamiina, Nagano 399-459, Japan

**Keywords:** Animal Welfare, Farmers′ Benefits, Milk Yield, Outdoor Access, Tethered Cows

## Abstract

**Objective:**

We investigated the effects of outdoor access for one-hour per day (ODA) on milk yield and composition and behaviors of tethered dairy cows.

**Methods:**

Eleven all-day tethered dairy cows were treated with ODA for two weeks. To evaluate the effect of ODA on milk yield, we first calculated the average daily milk yield of each cow for three days during two weeks before the ODA, three days before the ODA, three days at the end of the ODA, and three days during two weeks after the ODA. We then compared the milk yield change during the ODA with that for two weeks before and two weeks after the ODA. The effects of ODA on milk compositions and behaviors were evaluated by comparing the average values for each composition and behavior for the three days before the ODA and the last three days of the ODA.

**Results:**

The decrease of milk yield during the two weeks of ODA was significantly higher than that during the two weeks before ODA (p<0.01). The milk fat rate was significantly higher during ODA than before ODA (p<0.05). Lactose rate was significantly lower during ODA than before ODA (p<0.05). The concentrations of milk urea nitrogen, ketone bodies, and free fatty acids in the milk were significantly higher during ODA than before ODA (p<0.05). The mean total duration per day of lying during ODA was significantly lower than that before ODA (p<0.05). The walking steps per one-hour outdoor access were 158.4±54.7. The social behavior during the one-hour outdoor access of the 11 cows was 53 times/h/herd.

**Conclusion:**

Our results suggested that ODA promotes the expression of normal behavior in dairy cows, but even one hour of ODA decreases milk production in cows, which may drop producers’ profits without some financial supplementation.

## INTRODUCTION

A tethering system makes it easier to manage cows than grazing and free-stalls, and can prevent agonistic behavior and feed competition between cows. In dairy farming, cows fed in tethering systems produce more milk than those fed in free-stall and grazing systems [1,2]. Therefore, the tethering system is widely used worldwide [3–6]. However, the tethering system results in decreased expression of normal behavior [7], increased expression of abnormal behavior [8], and deterioration of the health status of dairy cows [9], thus reducing animal welfare (AW).

Tethered cows introduced for outdoor access have been known to have higher AW levels than those tethered throughout the day [10]. Outdoor access for one-hour per day (ODA) can promote normal behavior expression [11], reduce nipple infection [12], and enhance the immune function of dairy cows [13]. It has been suggested that outdoor access for ODA is one of the measures used to increase AW levels in tethered cows. However, their effects on milk yield and quality have not been clarified. A previous study suggested that tethered cows introduced to outdoor access produce less milk than tethered cows that spend the whole day tethered [14]. In their study, the time and frequency of outdoor access were unclear. Another study showed that extreme walking in cows reduced milk yield and altered milk composition [15]. The methods used in their study involved forcing the cows to walk. This forced walking, may cause stress to the cow, and may also create a labor burden on the farmer.

In this study, we adopted an outdoor access method in which the farmer dose not drive cows but treats them gently and evaluated the effects of one-hour daily outdoor access on milk yield and composition, and behaviors of tethered dairy cows. The one-hour daily outdoor access treatment period in this study was set at two weeks. The reason for setting this period is that when cattle changed from grazing to indoor tethering, urinary cortisol levels were higher in the first week and remained at the same level as during grazing from the second week [16]. This finding suggests that the response of the cattle to environmental changes may have weakened during the second week. In addition, the milk yield and composition analysis in this study used data collected from cows over 40 days of lactation. The reason for this is that the milk yield and composition vary with the number of lactation days. Holstein cows in Japan have an obvious peak in milk yield at approximately 40 days of lactation, which then decreased gradually, but milk fat, non-fat solids and milk protein were characterized by a marked decrease at the 40th day of lactation, followed by a moderate increase [17].

## MATERIALS AND METHODS

This study was performed in accordance with the Animal Experimental Regulations of Shinshu University (Approval No. 020031). Additionally, this study was also conducted in accordance with the rules and regulations of the Basic Guidelines for the Conduct of Animal Experiments at Research Institutes under the Jurisdiction of the Ministry of Agriculture, Forestry and Fisheries, Japan.

### Animals

We used 17 Holstein dairy cows reared on a farm at the Livestock Research Institute, Forestry and Fisheries Research Center, Toyama Prefectural Agriculture. Data from 11 cows with a lactation period of 104 to 302 days was used to analyze milk yield and composition and behaviors (Table 1).

### Breeding facility and management

**Figure 1:**
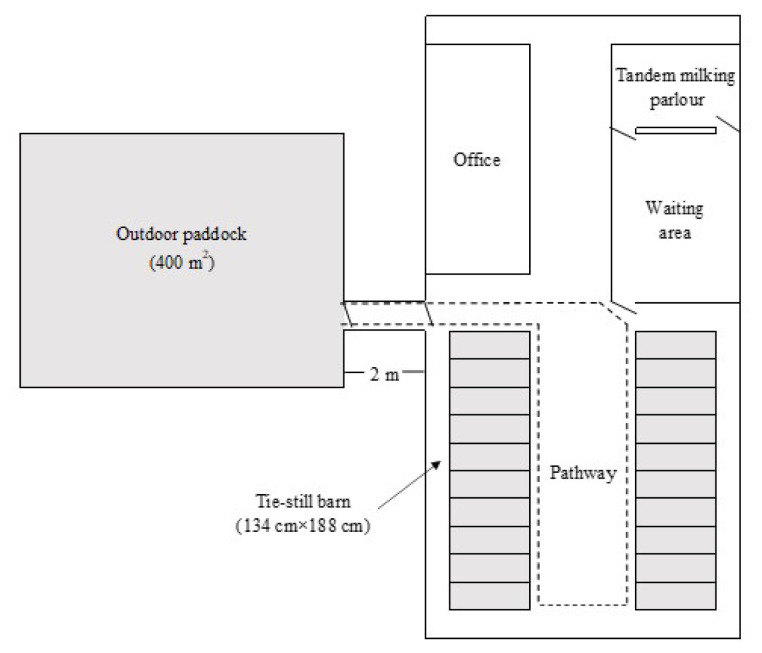
Schematic diagram of the cow barn and outdoor paddock.

The breeding facility consisted of a tie-rail-type stall, tandem milking parlor, milking waiting area, and an outdoor paddock (flat soil ground) 2 m away from the tethered place (Figure 1). The alignment of cows in the barn was so that their rounds were facing each other. During the experiment, there was no grass in the outdoor paddock.

In accordance with the Japanese feeding standards for dairy cattle [18], the cows were continuously fed a total mixed ration (TMR) (dry matter [DM], 44.4%; crude protein [CP], 5.5%; crude ash [CA], 9.8%; neutral detergent fiber [NDF], 57.0%; acid detergent fiber [ADF], 34.4%; non-fiber carbohydrate [NFC], 24.8%; ether extract [EE], 2.9%; total digestible nutrients [TDN], 60.2%) based on Phleum pratense and Medicago sativa hay. Daily TMR per cow was 31.4 kg. In addition, a commercial concentrated diet (DM, 87.5%; CP, 20.8%; CA, 5.9%; NDF, 21.5%; ADF, 9.8%; NFC, 45.6%; EE, 6.0%; TDN, 87.0%) was provided five times daily (5:00, 9:00, 11:30, 16:20, 20:00) using automatic feeding machines. The average daily concentrate diet per cow was 8.7 kg. The roughage-to-concentrate ratio was 59:41. Feeding was conducted in tie-rail-type stalls. The cows had free access to mineral salts and water. The cows were raised in tethering but were released from tethering and moved to the milking parlor at 5:00 and 15:00 every day. Thus, together with the outdoor access time described below, the one-day average release time (±standard deviation) per cow was 135.7±24.6 minutes.

### Experimental design

This study was conducted between October 31 and November 17, 2022. The average daily outside temperature during the experimental period ranged from 10.0°C to 18.1°C. After milking in the morning, the cows were not forcibly driven away, moved voluntarily, and were then released freely into the outdoor paddock adjacent to the barn between 7:30 and 8:30 (ODA period). All experiments were conducted in non-rainy weather because cows do not prefer to be outdoors on rainy days [19]. In addition, no outdoor access was provided to the estrous cows.

### Effect of outdoor access for one-hour per day on milk yield

When comparing the milk yield at the end with that at the beginning of the ODA treatment, the change in milk yield along with lactation days might have an impact on the results, and it may not be possible to correctly evaluate the effect of ODA. Therefore, we first calculated the average daily milk yield of each cow for three days during two weeks before the ODA, three days before the ODA, three days at the end of the ODA, and three days during two weeks after the ODA. Then, we compared the milk yield change during the ODA with the milk yield change two weeks before and two weeks after the ODA to evaluate the effect of ODA on milk yield.

### Effect of outdoor access for one-hour per day on milk composition

Milk composition was analyzed using milk from each cow in the afternoon (15:00) three days before the ODA and the last three days of the ODA. Milk fat, non-fat solids, milk protein, lactose, somatic cell count, milk urea nitrogen (MUN), ketone bodies, and free fatty acids were measured as specified by the Hokuriku Federation of the Dairy Cooperative Association. The effects of ODA on each item were evaluated by calculating and comparing the average values for each measurement item for the three days before the ODA and the last three days of the ODA.

### Effect of outdoor access for one-hour per day on behaviors

Behavioral observations were also made during three days before the ODA and during the last three days of the ODA. Surveillance cameras (wtw-dehp582e-4tb; Wireless Tsukamoto, Japan) were used to record the behavior of the cows continuously. The observation items included lying down, walking, and social behaviors. Behavioral data were first collected for the total duration and bout frequency per day (24 h) and mean bout duration in lying and then calculated as an average for each cow for the three days before the ODA and the last three days of the ODA. The effect of ODA on behavior was evaluated by comparing the average behavioral data for each cow for the three days before the ODA and the last three days of the ODA. Walking and social behaviors were observed during the outdoor access time on the last day of the ODA. Because the distance of one step of the cow was approximately 1.2 m, the walking distance when released into the outdoor space was calculated using this value. In addition, observed social behaviors were gently pushed through head-to-head interactions.

### Statistical analysis

Statistical analyses were performed using R version 4.1.0 [20]. First, the Shapiro test was used to analyze the normality of all data in the analysis items. Then, the effects of one-hour-ODA on milk yield and components, and behavior were analyzed as follows: if the data were normally distributed, they were analyzed using the paired t-test; if the data were not normally distributed, they were analyzed using the Exact Wilcoxon-Pratt Signed-Rank test.

## RESULTS

### Effect of outdoor access for one-hour per day on milk yield

**Figure 2:**
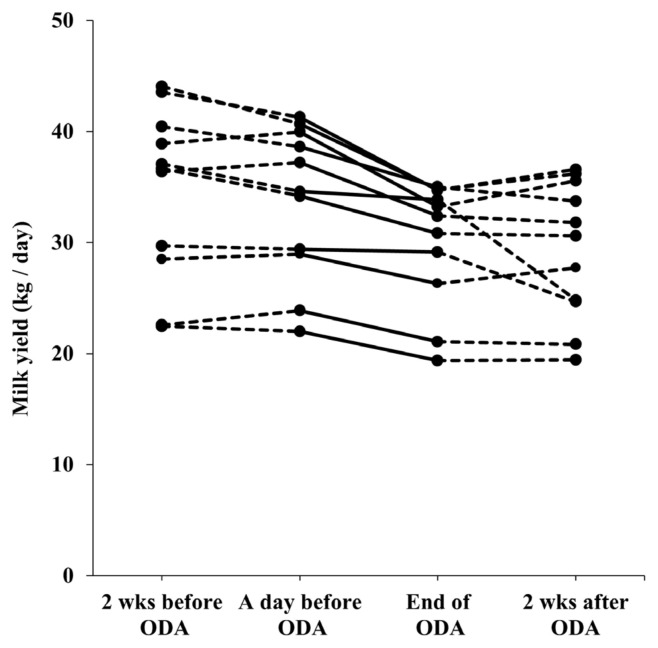
Changes in milk yield from two weeks before the start to two weeks after the end of the ODA (n = 11). ODA, outdoor access for one-hour per day.

**Figure 3:**
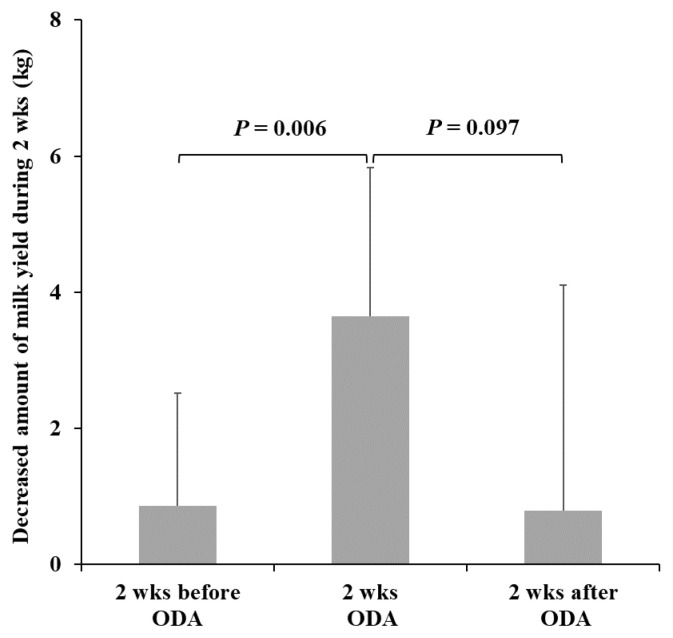
Comparison of milk yield change during the experiment with that of two weeks before and two weeks after the ODA (n = 11). ODA, outdoor access for one-hour per day; wks, weeks.

The daily milk yield (mean±standard error [SE]) of the cows was 34.6±7.7 kg/d for three days during two weeks before the ODA, 33.7±6.8 kg/d for three days before the ODA, 30.1± 5.6 kg/d for the last three days of the ODA, and 29.3±6.2 kg/d for three days during two weeks after the ODA. Milk yield decreased dynamically in all periods (in the middle and late lactation cows), but decreased significantly during ODA (Figure 2). The reduced milk yield (mean±SE) during two weeks of ODA was 3.7±2.2 kg, which was significantly higher than that during the two weeks before ODA (t = −3.25, df = 10, p = 0.006, Figure 3). Compared to the decreased milk yield during two weeks after ODA, it also showed a more significant trend (t = 1.83, df = 10, p = 0.097, Figure 3). In addition, the reduced milk yield during two weeks of ODA of 6 cows in the middle lactation (104 to 161 days postpartum) tended to be lower than that during the two weeks before ODA (t = −2.34, df = 5, p = 0.066). The reduced milk yield during two weeks of ODA of 5 cows in the late lactation (198 to 302 days postpartum) also tended to be lower than that during the two weeks before ODA (t = −2.47, df = 4, p = 0.069).

### Effect of outdoor access for one-hour per day on milk composition

The milk composition values before and during ODA are shown in Table 2. The milk fat rate was significantly higher during ODA than before ODA (t = −2.63, df = 10, p = 0.025). Milk protein rate tended to be higher during ODA than before ODA (t = −1.90, df = 10, p = 0.087). The lactose rate was significantly lower during ODA than before ODA (t = 2.34, df = 10, p = 0.041). Concentrations of MUN, ketone bodies and free fatty acids in milk were significantly higher during ODA than before ODA (MUN: t = −3.12, df = 10, p = 0.011; ketone bodies: t = −3.32, df = 10, p = 0.008; free fatty acids: t = −3.34, df = 10, p = 0.004).

### Effect of outdoor access for one-hour per day on behaviors

The mean total duration and bout frequency per day, and the mean bout duration of lying before and during ODA are shown in Table 3. The mean total duration per day of lying during ODA was significantly lower than that before ODA (Z = 2.22, p = 0.024; Table 3). The walking steps number per one-hour outdoor access was 158.4±54.7 (mean±SE). The walking distance of the cow was 190 m per one-hour outdoor access. The frequency of social behavior during the one-hour outdoor access of the 11 cows was 53 times/h/herd. These behaviors were not observed during the same pre-ODA period, because they were next to each other during tethering.

## DISCUSSION

Tethered cows introduced for outdoor access produce less milk than cows that spend all day tethered [14]. However, in their study, the duration and frequency of outdoor access were unclear. In present study, a short duration of ODA treatment also reduced the milk yield of dairy cows. In addition, the cows in the middle and late lactation responded similarly to ODA. ODA promotes walking in tethered cows, thereby improving their AW [11]. However, although walking is one of the main behaviors of dairy cows, it also increases their energy consumption and decreases milk yield [15]. These changes in milk yield are due to the lack of additional supplement intake by milking cows to compensate for increased energy requirements during walking [15]. Thus, many studies have focused on the effects of walking distance (1 to 12.8 km) on milk yield in dairy cows [15,21–23]. However, in these studies, walking was promoted in cows; because it was a mandatory exercise, the cows may have been stressed. In this study, the cows were allowed to act freely for the one-hour of ODA. However, the spontaneous walking distance in this study was much shorter than those reported in previous studies. This suggests that the effects of ODA on milk yield may be related to factors other than walking. However, the reason for this requires further investigation.

Loberg et al [11] reported that there was no difference in milk yield between cows fed ODA and those tethered all day. They divided the cows into four groups (13 cows each): exercise every day, two days per week, one day per week, and no exercise. The effects of the feeding treatments on milk yield were analyzed considering the effects of cow age and lactation stage. Lactation was divided into four stages: 1 to 3 months after parturition, 4 to 8 months after parturition, dry cows, and heifers. However, milk yield varies with the number of days of lactation, with a clear peak at approximately 40 days, followed by a decrease [1,17]. Therefore, in the study by Loberg et al [11], owing the long duration of each lactation stage, the analysis of the effect of feeding treatment on milk yield may inevitably be influenced by the difference in individual lactation days. In present study, we analyzed the difference in milk yield after 40 days of lactation to exclude the effect of lactation days on milk yield.

In the present study, milk fat rate was increased by ODA. This result is consistent with previous studies that investigated the effects of walking on milk quality [15,24]. The increase in milk fat rate by ODA may be due to a decrease in milk yield [15]. In addition, the milk protein rate in ODA tended to increase in this study. This may also be attributed to decreased milk yield [15]. On the other hand, the lactose rate was decreased by ODA treatment in present study. Daytime outdoor access has been reported to reduce lactose rate in milking eyes [25]. Moreover, the lactose content of dairy cows under pasture-raising conditions is lower than that of indoor-raised cows [26]. However, the cause has not yet been elucidated, and further investigation is required.

ODA increased MUN concentration in this study. MUN concentration can be used to monitor the nutritional status of dairy cows during lactation [27]. An increase in MUN may indicate that cows consume excessive protein [28]. It suggests that ODA might promote protein digestion in dairy cows. In addition, ketone bodies and free fatty acids were increased by ODA. During the early stages of lactation, feed intake decreases with an increase in milk yield, resulting in a negative energy balance in cows. The animal draws on body fat reserves to provide the energy needed for milk production, thereby increasing the concentrations of free fatty acids and ketone bodies [29]. In this study, the cows subjected to ODA may have consumed more energy than those left tethering, which may have led to an increase in the concentration of free fatty acids and ketone bodies.

The AW grade evaluation criteria recommend measuring the lying time of cows [30]. Our results showed that the mean total duration per day of lying during ODA was significantly lower than that before ODA, but the normal behaviors such as walking and social behaviors increased. Similar results have been reported in dry cows [13]. This suggests that ODA may have promoted normal behavioral expression, except for lying, owing to the increased available space for tethered cows gained through outdoor access for one-hour [13]. The expression of social behavior is used as an evaluation criterion for AW levels [10]. In addition, it has been reported that head butts in tethered cows with outdoor exercise is more frequent than those in cows that are left tethering all day [10]. However, increased agonistic behavior may indicate uncomfortable or stressful situations [31]. In this study, observed social behaviors were gently pushed through head-to-head interactions. This is probably because the cows were together during daily milking or during the two weeks outdoor access period, so they did not see each other as enemies and had lower levels of aggressive behavior.

Because milk yield varies depending on various environmental factors, this study used all milking cows housed in the same environment. Therefore, this study determined the extent of milk yield decrease during, before and after ODA's introduction. However, because the study was conducted in the fall when the weather was relatively mild, further research should be conducted in different seasons and outdoor paddock sizes.

## CONCLUSION

ODA resulted in a decrease in milk yield and a change in the composition of dairy cows in the tethering system. These results suggest that ODA can lead to a significant reduction in benefits to farmers. However, ODA may have promoted normal behavior expression, owing to the increased available space for tethered cows gained through outdoor access for one-hour, towards that typically found in grazing or free barn feeding systems. This is highly effective in improving the AW of tethered cows. Our results provide useful scientific knowledge on the dissemination of AW in tethering dairy cattle production sites. However, the balance between AW improvement and production efficiency remains problematic. This may require government support or incur a consumer burden. Further studies are necessary to confirm this hypothesis.

## Figures and Tables

**Table 1:** Basic information of the tested cows in this study

Cow No.	Age	Weight (kg)	Parity	Postpartum days at the start of the experiment
288	2	550	1	21
278	3	586	2	37
261	4	618	3	41
285	2	502	1	44
287	2	574	1	44
243	5	716	3	52
256	4	676	4	104
284	2	560	1	123
275	3	700	2	127
271	3	638	2	128
269	4	680	2	132
232	6	712	5	161
272	3	656	2	198
270	3	598	2	218
283	2	592	1	267
282	2	594	1	274
280	3	530	1	302

Data from cows with a lactation period of 104 to 302 days was used to analyze milk yield and composition and behaviors.

**Table 2:** Effect of outdoor access for one-hour per day (ODA-one-hour) on milk composition of tethered dairy cows (n = 11)

Items	Before ODA	ODA-one-hour	t or Z	df	p-value
Fat (%)	4.30±0.48	4.52±0.69	−2.63	10	0.025
Solids not fat (%)	8.91±0.19	8.90±0.25	0.19	10	0.854
Protein (%)	3.37±0.20	3.42±0.24	−1.90	10	0.087
Lactose (%)	4.54±0.07	4.49±0.08	2.34	10	0.041
Somatic cell count (×10^3^ cells/mL)	17.00 (10.17–24.34)	23.33 (13.00–33.00)	−0.85		0.426
Milk urea nitrogen (mg/dL)	7.23±1.27	8.20±1.14	−3.12	10	0.011
Ketone bodies (mM/L)	0.08±0.02	0.10±0.03	−3.32	10	0.008
Free fatty acid (mmol/100 g)	1.08±0.33	1.34±0.43	−3.34	10	0.004

Values are means±standard error for parametric statistics (t-values) and are medians (inter-quartile ranges) for non-parametric statistics (Z-values).

**Table 3:** Effect of outdoor access for 1 h per day (ODA-1 h) on lying of tethered dairy cows (n = 11)

Items	Tethering	ODA-1 h	Z	p-value
Total duration (min/d)	738.40 (685.90–753.25)	695.50 (662.10–743.80)	2.22	0.024
Bout frequency (bouts/d)	10.70 (9.85–12.65)	10.00 (9.15–12.00)	0.71	0.502
Mean bout duration (min)	72.20 (56.95–75.55)	69.20 (61.70–75.05)	0.53	0.638

Values are medians (inter-quartile ranges) for non-parametric statistics.
